# Cholestenoic acid as endogenous epigenetic regulator decreases hepatocyte lipid accumulation in vitro and in vivo

**DOI:** 10.1152/ajpgi.00184.2023

**Published:** 2023-11-14

**Authors:** Yaping Wang, Williams M. Pandak, Phillip B. Hylemon, Hae-Ki Min, John Min, Michael Fuchs, Arun J. Sanyal, Shunlin Ren

**Affiliations:** Department of Internal Medicine, Virginia Commonwealth University, Richmond, Virginia, United States;; McGuire Veterans Affairs Medical Center, Richmond, Virginia, United States

**Keywords:** bile acids, cholestenoic acid, cholesterol and lipids metabolism, DNA methyltransferase, oxysterol

## Abstract

Cholestenoic acid (CA) has been reported as an important biomarker of many severe diseases, but its physiological and pathological roles remain unclear. This study aimed to investigate the potential role of CA in hepatic lipid homeostasis. Enzyme kinetic studies revealed that CA specifically activates DNA methyltransferases 1 (DNMT1) at low concentration with EC_50_ = 1.99 × 10^−6^ M and inhibits the activity at higher concentration with IC_50_ = 9.13 × 10^−6^ M, and specifically inhibits DNMT3a, and DNMT3b activities with IC_50_= 8.41 × 10^−6^ M and IC_50_= 4.89 × 10^−6^ M, respectively. In a human hepatocyte in vitro model of high glucose (HG)-induced lipid accumulation, CA significantly increased demethylation of ^5m^CpG in the promoter regions of over 7,000 genes, particularly those involved in master signaling pathways such as calcium-AMPK and 0.0027 at 6 h. RNA sequencing analysis showed that the downregulated genes are affected by CA encoding key enzymes, such as PCSK9, MVK, and HMGCR, which are involved in cholesterol metabolism and steroid biosynthesis pathways. In addition, untargeted lipidomic analysis showed that CA significantly reduced neutral lipid levels by 60% in the cells cultured in high-glucose media. Administration of CA in mouse metabolic dysfunction-associated steatotic liver disease (MASLD) models significantly decreases lipid accumulation, suppresses the gene expression involved in lipid biosynthesis in liver tissues, and alleviates liver function. This study shows that CA as an endogenous epigenetic regulator decreases lipid accumulation via epigenetic regulation. The results indicate that CA can be considered a potential therapeutic target for the treatment of metabolic disorders.

**NEW & NOTEWORTHY** To our knowledge, this study is the first to identify the mitochondrial monohydroxy bile acid cholestenoic acid (CA) as an endogenous epigenetic regulator that regulates lipid metabolism through epigenome modification in human hepatocytes. The methods used in this study are all big data analysis, and the results of each part show the global regulation of CA on human hepatocytes rather than narrow point effects.

## Graphical abstract

**Figure:**
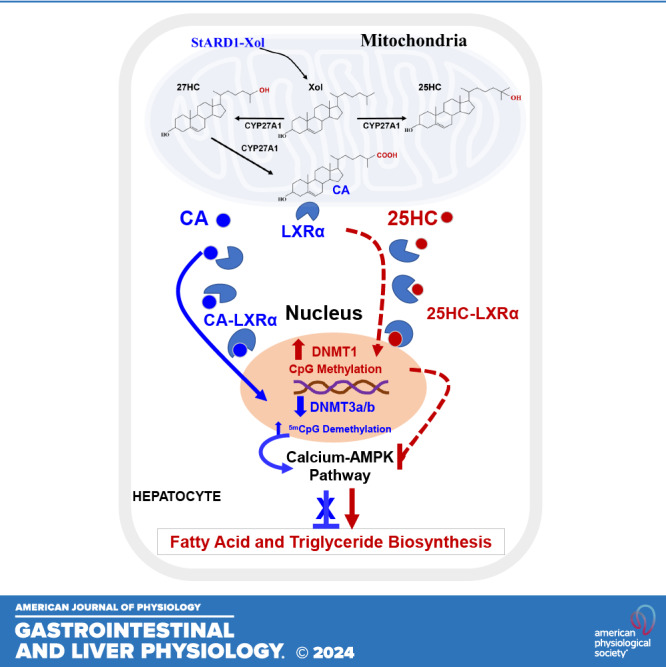

## INTRODUCTION

There are two hepatic pathways for bile acid biosynthesis: “classic” and “acidic” pathways (1). The classic pathway is initiated by the microsomal cholesterol 7-hydroxylase (CYP7A1) and followed by sterol 12-hydroxylation to produce cholic acid. The “acidic” pathway of bile acid biosynthesis is initiated by mitochondrial sterol 27-hydroxylase (CYP27A1) followed by 7α-hydroxylation by CYP7B1 (2). Interestingly, CYP27A1 can synthesize 27-hydroxycholesterol (27HC), 25-hydroxycholesterol (25HC), and cholestenoic acid (CA), which have been shown to be naturally occurring ligands for liver X receptors (LXRs) (3, 4). 25HC and 27HC have been reported to play important roles in lipid metabolism and cell apoptosis. These oxysterols regulate the expression of many genes encoding enzymes involved in cholesterol biosynthesis, lipid metabolism, and cell proliferation (5–7). Increased CYP27A1 enzyme activity in peripheral tissues has been reported to downregulate cholesterol biosynthesis (8). Gene knockout of CYP7B1 results in accumulation of these oxysterols in cells (8–11). Additional evidence has shown that these oxysterols are associated with a wide variety of cellular functions (12). 25HC and 27HC can be sulfated at the 3β-hydroxyl group and counteract the effects of oxysterols by decreasing lipid biosynthesis, suppressing inflammatory responses, and promoting cell survival (5, 13–27). The relative concentrations of cellular oxysterols and oxysterol sulfates may function as a “cellular rheostat” by regulating promoter activities via a methylation/demethylation mechanism. In this regard, 25HC and 27HC activate DNA methyltransferases 1 (DNMT1) with EC_50_ ∼3 × 10^−6^ M, whereas sulfated 25HC (25HC3S) and sulfated 27HC (27HC3S) significantly suppress DNMT activities with IC_50_ ∼4 × 10^−6^ M (27). CA is a cholesterol metabolite with a unique chemical structure containing a 26-carboxylic acid group. CA is the only primary bile acid that does not contain a 7-hydroxyl group (28). CA has been reported to be a biomarker of alveolar macrophages functional integrity and its cellular level decreases with increasing disease severity in patients with acute respiratory distress syndrome (ARDS) (29, 30). CA has also been reported to act as an endogenous γ-secretase modulator (GSM) within the brain and it has been hypothesized that increased levels of CA in the brain may help prevent Alzheimer’s disease (AD) (31). Its activation of biochemical signaling pathway(s) and mechanism of action is currently unknown although recent reports have shown that 25HC and 27HC are potent DNMT1 activators (27).

Epigenetic pathways regulating DNA methylation may play a major role in the regulation and coordination of global gene expression. Methylation at position 5 of cytosine (5-methylcytosine,^5m^C) in DNA is an important epigenetic modification that regulates gene expression (32). The methylation of cytosine in the CpG sequences located in the promoter region of genes is directly correlated with transcriptional activity, as it leads to chromatin condensation and gene silencing (33). Recently published literature has demonstrated that dysregulation of CpG methylation and gene expression is important in metabolism and homeostasis (34–36). It has been shown that cytosine methylation is carried out by DNMT-1, 3a/3b (37, 38). However, how these DNA methyltransferases are regulated, and their endogenous ligands, are currently unclear.

In the present study, we provide evidence that CA regulates DNMT1 and suppresses DNMT3a/3b, subsequently decreasing the expression of 3-hydroxy-3-methylglutaryl-CoA reductase (HMGR), proprotein convertase subtilisin/kexin type 9 (PCSK9), and fatty acid synthase (FAS). The results suggest that CA may play an important role in the maintenance of intracellular cholesterol and lipid homeostasis in hepatocytes.

## MATERIALS AND METHODS

### Materials

Cell culture reagents and supplies were purchased from Gibco BRL (Grand Island, NY). HepG-2 cells were obtained from the American Type Culture Collection (Rockville, MD). The reagents for quantitative reverse transcription PCR (RT-qPCR) were from AB Applied Biosystems (Warrington, UK). The chemicals used in this study were obtained from Sigma Chemical Co. (St. Louis, MO) or Bio-Rad Laboratories (Hercules, CA). All solvents were obtained from Fisher (Fair Lawn, NJ) otherwise indicated.

#### Cell culture.

HepG-2 cells were cultured in DMEM medium supplemented with 10% heat-inactivated fetal bovine serum (FBS) and high glucose (HG, 4.5 g/L) at 37°C in a humidified atmosphere of 5% CO_2_.

### Extraction and Determination of DNA and mRNA Levels

After HepG-2 cells were cultured in DMEM medium with HG for 72 h followed by treatment with 20 µM CA for 0, 3, 6, 12, and 24 h, genomic DNA from 5 × 10^7^ cells were extracted using QIAamp DNA Mini Kit (QIAGEN, Hilden, Germany). Each 6-µg sample was sent to CD Genomics Co., Ltd. (New York) for analysis of whole genome bisulfite sequencing (WGBS). Total RNA was isolated using the Promega SV total RNA isolation system (Madison, WI) with DNase treatment. Each sample, 2 µg, was sent to CD Genomics Co., Ltd. (New York) for analysis of RNA sequencing. The same samples, 2 µg, were used for the first-strand cDNA synthesis as recommended by the manufacturer (Invitrogen, Carlsbad, CA). RT-qPCR was performed using SYBR Green as the indicator on the ABI 7500 Fast Real-Time PCR System (Applied Biosystems, Foster City, CA). Amplifications of β-actin or GAPDH were used as internal controls. Relative messenger RNA (mRNA) expression was quantified with the comparative cycle threshold (Ct) method using the primer set shown in Supplemental Table S1 and was expressed as 2^−ΔΔCt^ as described previously (39).

### Enzyme Kinetic Study of CA

The enzyme kinetic studies were carried out by Reaction Biology Company (Malvern, PA). For the DNMT1 activity assay, the substrate solution, 0.001 mg/mL of poly(dI-dC) (in 50 mM Tris·HCl, pH 7.5, 50 mM NaCl, 5 mM EDTA, 5 mM DTT, 1 mM PMSF, 5% glycerol, 0.01% Brij35, and 1% DMSO) was used. For the DNMT3a/3b activity assay, 0.0075 mg/mL Lambda DNA in 50 mM Tris·HCl, pH 7.5, 50 mM NaCl, 5 mM EDTA, 5 mM DTT, 1 mM PMSF, 5% glycerol, and 1% DMSO was used. The indicated DNMT1, DNMT3a, and DNMT3b were added to the appropriate substrate solution and gently mixed. Amounts of CA ranging from 5.08*e*-09 to 0.0001 M in DMSO were added to the reaction mixture by using Acoustic Technology (Echo 550, LabCyte, Inc., Sunnyvale, CA). The mixtures were first incubated for 15 min, and then adenosyl-l-methionine, *S*-[methyl-3H] (3H-SAM) was added to the reaction mixture to initiate the reaction, and the mixture was incubated for 60 min at 30°C. After incubation, the reaction mixture was finally transferred to filter paper for detection of radioactivity counts.

### Analysis of Whole Genome Bisulfite Sequencing

The whole genome bisulfite sequencing (WGBS) was conducted by CD Genomic Company (NY). Each 1-μg sample of genomic DNA was fragmented by sonication to a mean size of ∼200–400 bp, and subsequently used for WGBS library construction using Acegen Bisulfite-Seq Library Prep Kit (Acegen) following the manufacturer’s instructions. The methylated adapter-ligated DNAs were purified using 0.8 × Agencourt AMPure XP magnetic beads and subjected to bisulfite conversion by ZYMO EZ DNA Methylation-Gold Kit (Zymo Research Corporation). The converted DNAs were then amplified using 25 μL of KAPA HiFi HotStart Uracil+ ReadyMix (2×) and 8-bp index primers with a final concentration of 1 μM each. The constructed WGBS libraries were then analyzed by Agilent 2100 Bioanalyzer, quantified by a Qubit fluorometer with Quant-iT dsDNA HS Assay Kit (Invitrogen), and finally sequenced on Illumina Hiseq X ten sequencer. After the preparation of the library, Qubit 2.0 and Agilent 2100 were used respectively to detect the concentration of the library and the Insert Size, and the effective concentration (>2 nM) of the library was quantitatively determined by Q-PCR to ensure the library quality.

Samples were sequenced using the Illumina HiSeq sequencing platform. Raw data generated on the sequencing platform contained a small percentage of low-quality data, which was then filtered to get high-quality data. Bsmap software was used to perform alignments of bisulfite-treated reads to a reference genome (GRCh37). Metilene software was used to identify differentially methylated regions (DMRs). DAVID software (https://david.ncifcrf.gov/) was used to test the statistical enrichment of DMR-related genes in the Gene Ontology (GO) and Kyoto Encyclopedia of Genes and Genome (KEGG) pathways.

### Transcriptional Profiling and Data Analysis

Total RNA was extracted and purified from HepG-2 cells using the SV total RNA isolation system (Promega, Madison, WI). Messenger RNA was purified from total RNA using poly-T oligo-attached magnetic beads. After fragmentation, the first strand cDNA was synthesized using random hexamer primers, followed by the second strand cDNA synthesis using either dUTP for directional library or dTTP for nondirectional library. The library was checked with Qubit and real-time PCR for quantification and bioanalyzer for size distribution detection. Quantified libraries will be pooled and sequenced on Illumina platforms, according to effective library concentration and data amount. Raw data (raw reads) of fastq format were first processed through in-house perl scripts. In this step, clean data (clean reads) were obtained by removing reads containing adapter, reads containing ploy-N, and low-quality reads from raw data. At the same time, Q20, Q30, and GC content of the clean data were calculated. All the downstream analyses were based on clean data with high quality. The clean data were aligned to reference genome (GRCh37) using Hisat2 v2.0.5 software. Differential expression genes (DEGs) were performed using the DESeq2 R package (1.20.0). DAVID software (https://david.ncifcrf.gov/) was used to test the statistical enrichment of DMR-related genes in the GO and KEGG pathways.

### Qualification and Quantification of Intracellular Neutral Lipids in Hepatocytes

#### Oil Red O staining.

HpG-2 cells were seeded on 22 × 22-mm glass coverslips in six-well plates. Cells were cultured in DMEM media with high glucose for 72 h, followed by treatment with 0, 2.5, 5, 10, 20, and 50 µM of CA for 24 h. The treated cells were fixed with 3.7% formaldehyde in phosphate-buffered saline (PBS) for 30 min, followed by three washes with PBS. The cells were stained with 0.2% Oil Red O in 60% isopropanol for 10 min and washed three times with PBS. The stained lipids were examined by microscopy as previously described (40).

#### Untargeted lipidomics analysis.

HepG-2 cells were cultured in DMEM medium with HG for 72 h followed by treatment with 20 µM CA for 24, 48, and 72 h. The cells were harvested with 500 µL 1× PBS, and sent to Creative Proteomics Co., Ltd. (New York) for untargeted lipidomics analysis. Samples were thawed and 1.5 mL of chloroform:methanol (2:1, vol/vol) were added to sample, vortexed for 1 min, and followed by sonication for 30 min at 4°C, and then centrifuged at 12,000 rpm, 4°C for 10 min, transferred the lower phase to a new tube, and dried under nitrogen. The dried extract was dissolved with 200 µL of isopropyl alcohol:methanol (1:1, vol/vol) and added 5 µL of LPC (12:0) as the internal standard. Finally, centrifuged at 12,000 rpm, at 4°C for 10 min; transferred the supernatant for LC-MS analysis. Separation is performed by Ultimate 3000 LC combined with Q Exactive MS (Thermo) and screened with ESI-MS. The LC system is composed of ACQUITY UPLC BEH C18 (100 × 2.1 mm × 1.7 µm) with Ultimate 3000 LC. The mobile phase is composed of solvent A [60% acetonitrile (ACN) + 40% H_2_O + 10 mM HCOONH_4_] and solvent B (10% ACN + 90% isopropyl alcohol + 10 mM HCOONH_4_) with a gradient elution (0–10.5 min, 30–100% B; 10.5–12.5 min, 100% B; 12.5–12.51 min, 100–30% B; 12.51–16.0 min, 30% B). The flow rate of the mobile phase is 0.3–1 mL·min^−1^. The column temperature is maintained at 40°C, and the sample manager temperature is set at 4°C. Mass spectrometry parameters in ESI+ and ESI− mode are listed as follows: ESI+: heater temperature 300°C; sheath gas flow rate, 45 au; aux gas flow rate, 15 au; sweep gas flow rate, 1 au; spray voltage, 3.0 kV; capillary temperature, 350°C; S-Lens RF level, 30%. ESI−: heater temperature 300°C, sheath gas flow rate, 45 au; aux gas flow rate, 15 au; sweep gas flow rate, 1 au; spray voltage, 3.2 kV; capillary temperature 350°C; S-Lens RF level 60%.

### Animal Studies

The animal studies were approved by the Institutional Animal Care and Use Committee of McGuire Veterans Affairs Medical Center and conducted in accordance with the Declaration of Helsinki, The Guide for the Care and Use of Laboratory Animals, and all applicable regulations. In this study, a Western diet-induced metabolic dysfunction-associated steatotic liver disease (MASLD) mouse model was used. To create the model, 8-wk-old C57BL/6J female mice were purchased from the Jackson Laboratory and fed a Western diet (TD.88137, Envigo) along with high glucose/fructose water [Western diet with sweet water (WDSW)] containing 23.1 g/L of fructose and 18.9 g/L of glucose for 12 wk. After the model was established, the mice were separated into three groups based on their weight. The control mice in each group received intravenous injection (iv) with vehicle (DMSO). In contrast, the treatment group mice were intravenously injected with 10 mg/kg of CA (dissolved in DMSO) with a total volume of less than 100 µL. During the treatment period, injections were administered every 2 days. All mice were housed under identical conditions in an aseptic facility with a 12-h light/12-h dark cycle and provided with free access to water and food (WDSW). Before euthanasia, the mice fasted overnight. Blood samples were collected, and the serum enzymatic activities of alkaline phosphatase (ALK), alanine aminotransferase (ALT), and aspartate aminotransferase (AST) were measured in the clinical laboratory at McGuire Veterans Affairs Medical Center.

#### Histological analysis.

The liver tissues of each mouse were collected and fixed in 10% paraformaldehyde in 0.1 M phosphate buffer at room temperature overnight. The regions of the specimens were standardized for all mice. The paraffin-embedded tissue sections (4 μm) were prepared by the Department of Pathology, School of Medicine, Virginia Commonwealth University, then deparaffinized and stained using a standard hematoxylin and eosin (H&E) method (13).

#### Quantification of hepatic lipids.

Total cholesterol and cholesterol ester were determined by biochemical kits according to the manufacturer’s instructions. Lipid contents were normalized to protein concentrations tested with protein quantitative assay kit (Bio-Rad). Briefly, 30 mg of liver tissues were homogenized, and then lipids were extracted with a mixture of chloroform and methanol (2:1, vol/vol). The filtered extracts, 0.2 mL, were evaporated to dryness and dissolved in 100 µL of isopropanol containing 10% of triton X-100 for total cholesterol and cholesterol esters assay (Wako Chemicals USA, Richmond, VA) (40).

### Statistics

Data are reported as the means ± standard deviation. Where indicated, data were subjected to *t* test analysis and determined to be significantly different if *P* < 0.05.

## RESULTS

### CA as a Ligand of DNMTs

**Figure 1.**
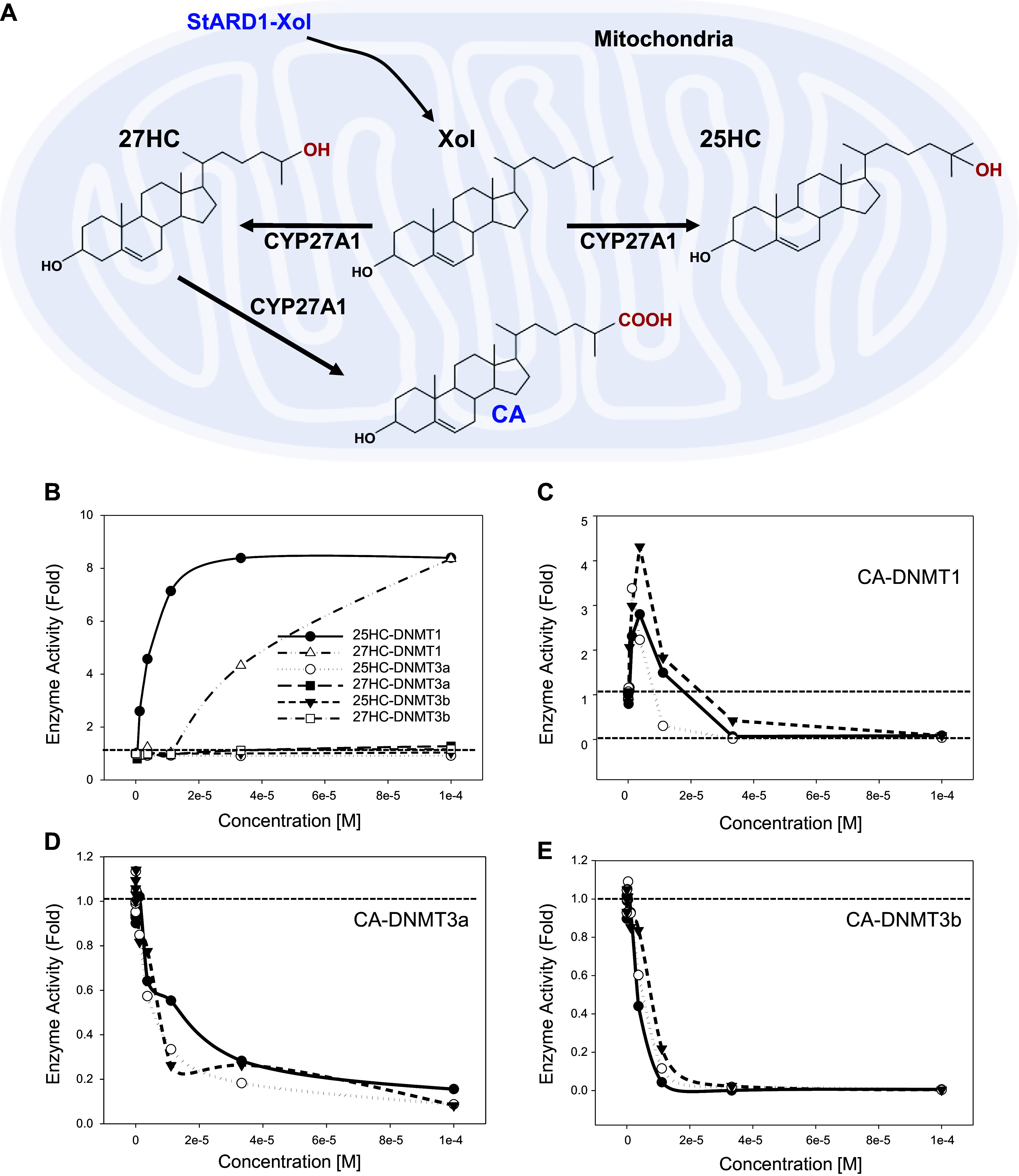
Biosynthesis and enzyme kinetic study of cholestenoic acid (CA). *A*: biosynthesis of 25-hydroxycholesterol (25HC), 27-hydroxycholesterol (27HC), and CA in mitochondria. *B*: concentration (0–0.0001 M)-dependent effect of 25HC and 27HC, on DNMT1/DNMT3a/DNMT3b enzymatic activity. *C*: effects of CA on DNMT1 enzymatic activity (EC_50_ = 9.13 ± 0.66 × 10^−6^ M; means ± SD; *n* = 3). *D*: effects of CA on DNMT3a enzymatic activity (IC_50_ = 8.41 ± 4.53 × 10^−6^ M; means ± SD; *n* = 3). *E*: effects of CA on DNMT3b enzymatic activity (IC_50_ = 4.89 ± 1.82 × 10^−6^ M; means ± SD; *n* = 3). Each point represents an individual data are pooled from three independent experiments.

27HC, 25HC, and CA biosynthesized in mitochondria have been previously reported as naturally occurring ligands for LXRs (Fig. 1*A*). 27HC and 25HC play important roles in lipid metabolism, inflammatory responses, and cell apoptosis (6, 41, 42). Our previous report showed that 25HC and 27HC both activate DNMT1 activity, and regulate lipid metabolism and cell apoptosis through DNA methylation (27). Enzyme kinetic effects of 25HC and 27HC on DNMT activity are summarized in Fig. 1*B*. To determine if CA affects DNA methyltransferase activity, recombinant DNMT1, DNMT3a, and DNMT3b were used for enzyme kinetic studies. The results demonstrated that CA specifically activates DNMT1 at low concentration with EC_50_ = 1.99 ± 0.72 × 10^−6^ M and inhibits activity at higher concentration with IC_50_ = 9.13 ± 0.66 × 10^−6^ M (Fig. 1*C*), and specifically inhibits DNMT3a, and DNMT3b activities with IC_50_= 8.41 ± 4.53 × 10^−6^ M and IC_50_= 4.89 ± 1.82 × 10^−6^ M, respectively, as shown in Fig. 1, *D* and *E*. As a positive control, *S*-adenosyl homocysteine inhibited DNMT1 activity by 95% at 1 µM, as previously reported (28). The results demonstrated that CA is a potent inhibitor of DNMT3a/3b and DNMT1 at high concentrations but as an activator of DNMT1 at low concentrations.

### Effects of CA on the Whole Genome-Wide DNA Methylation in Human Hepatocytes

**Figure 2.**
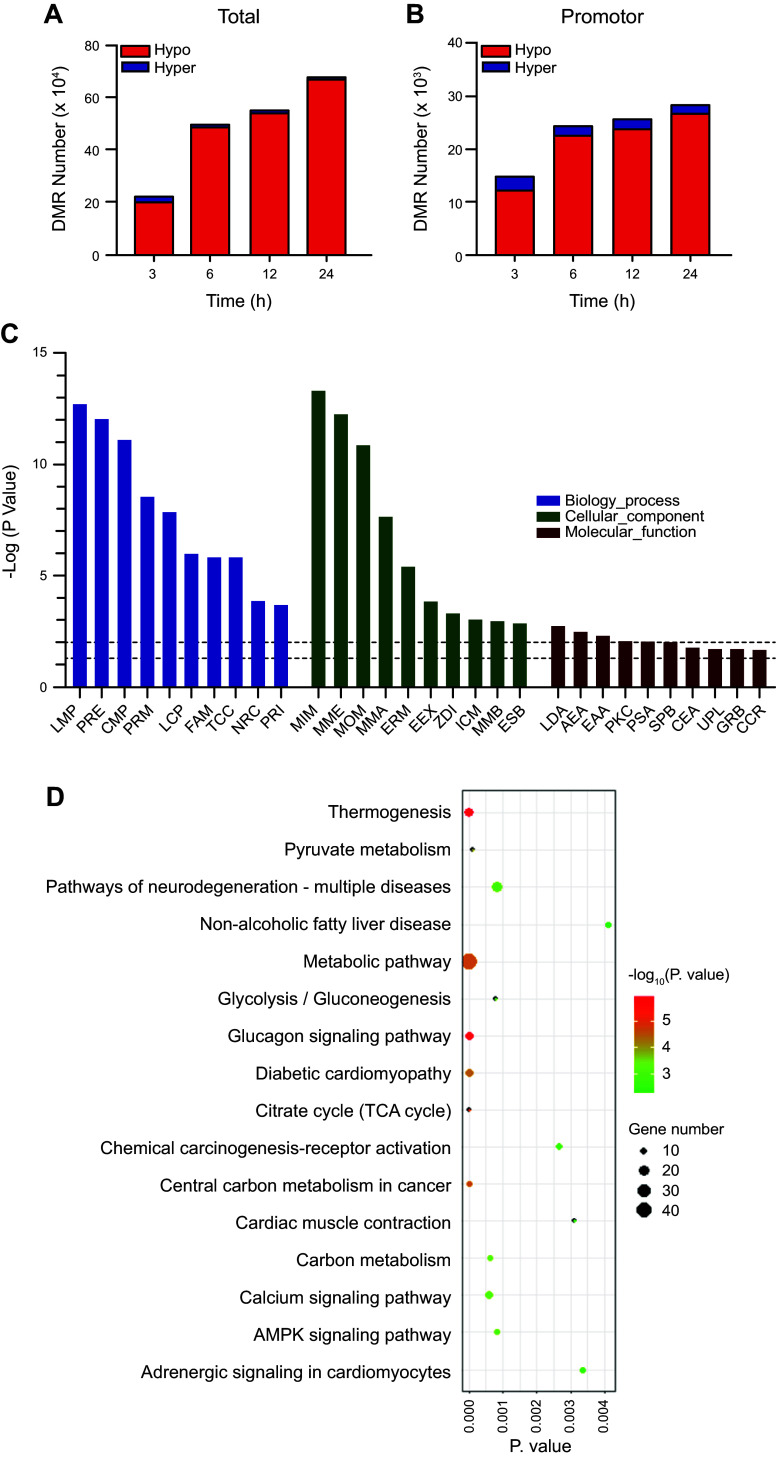
Effects of cholestenoic acid (CA) on DNA methylation in hepatocytes using whole genome bisulfite sequencing (WGBS) analysis. HepG-2 cells were cultured in high glucose (HG) medium for 72 h and followed by treatment with 20 µM CA treatment for 0, 3, 6, 12, and 24 h. One microgram of genomic DNA was used to prepare libraries. *A*: number of differential methylated regions (DMRs) in whole genome. *B*: number of DMRs in promoter regions. *C*: top terms (*P* < 0.05) of Gene Ontology (GO) analysis, enriched in hypomethylated DMRs in promoter regions. LMP, lipid metabolic process; PRE, positive regulation of ERK1 and ERK2 cascade; CMP, carbohydrate metabolic process; PRM, positive regulation of MAPK cascade; LCP, lipid catabolic process; FAM, fatty acid metabolic process; TCC, tricarboxylic acid cycle; NRC, negative regulation of cell growth; PRI, positive regulation of interleukin-6 production; MIM, mitochondrial inner membrane; MME, mitochondrial membrane; MOM, mitochondrial outer membrane; MMA, mitochondrial matrix; ERM, endoplasmic reticulum membrane; EEX, extracellular exosome; ADI, Z disc; ICM, integral component of mitochondrial inner membrane; MMB, membrane; ESP, extracellular space; LDA, l-lactate dehydrogenase activity; AEA, 1-alkyl-2-acetylglycerophosphocholine esterase activity; EAA, enzyme activator activity; PKC, protein kinase A catalytic subunit binding; PSA, protein self-association; SPB, S100 protein binding; CEA, cysteine-type endopeptidase activator activity involved in apoptotic process; UPL, ubiquitin protein ligase binding; GRB, GABA receptor binding; CCR: complement component C5a receptor activity. *D*: top significantly (*P* < 0.05) enriched Kyoto Encyclopedia of Genes and Genome (KEGG) pathways of promoter region with hypomethylated DMRs.

To determine the possible cellular functions of ^5m^CpG demethylation, HepG-2 cells were treated with 20 µM CA for 0, 3, 6, 12, and 24 h and harvested for the construction of bisulfite-treated genomic DNA libraries. In these libraries, more than 88% of bases have scores >Q30 for single and paired-end reads. The depth and density of sequencing were enough for a high-quality genome-wide methylation analysis. Moreover, the efficiencies of bisulfite conversion, represented by lambda DNA to the libraries, were over 99%, providing reliable and accurate results for the WGBS (Supplemental Table S2). CpG methylation and demethylation are well documented to relate to gene expression (35). A total of 227,565 DMRs were detected at 3 h following CA treatment (11,818 were hypermethylated, and 215,747 were hypomethylated) from the libraries from the cells that were treated with 20 µM CA compared with vehicle control. Moreover, at 6 h 508,222 (10,537 were hypermethylated, and 497,685 were hypomethylated), at 12 h 559,574 (14,658 were hypermethylated, and 544,916 were hypomethylated), and at 24 h 687,859 (8,808 were hypermethylated, and 679,051 were hypomethylated) DMRs were detected following CA treatment (Fig. 2*A*). Further analysis of DMRs showed that 14,754 DMRs (2,323 were hypermethylated, and 12,431 were hypomethylated) were in promoter region at 3 h, 24,370 (1,749 were hypermethylated, and 22,621 were hypomethylated) at 6 h, 25,704 (1,891 were hypermethylated, and 23,813 were hypomethylated) at 12 h, 28,356 (1,594 were hypermethylated, and 26,762 were hypomethylated) at 24 h (Fig. 2*B*). To analyze the function of hypomethylated DMRs in promoter regions, 24,370 DMRs that were treated with CA for 6 h were enriched into GO and KEGG database. The results showed that the major GO processes are lipid metabolism-related, including lipid metabolic process (LMP), positive regulation of ERK1 and ERK2 cascade (PRE), carbohydrate metabolic process (CMP), positive regulation of MAPK cascade (PRM), lipid catabolic process (LCP), and fatty acid metabolic process (FAM) (Fig. 2*C*). For KEGG database, these DMRs were significantly enriched into metabolic signaling pathways, including calcium, AMPK, nonalcoholic fatty liver disease (NAFLD), chemical carcinogenesis-receptor activation, and glucagon signaling pathways (Fig. 2*D*). Among these pathways, calcium and AMPK signaling are hypothesized to be master pathways regulating cell survival, antioxidants, antiapoptosis, energy metabolism, and lipid homeostasis (43–46). CA increased demethylation of ^5m^CpG in promoter regions of 13 genes involved in calcium signaling pathway (Table 1), nine genes involved in NAFLD pathway (Table 2), nine genes involved in AMPK signaling pathway (Table 3), 12 genes involved in glucagon signaling pathway (Table 4), and nine genes involved in chemical carcinogenesis receptor activation pathway (Table 5). The chromosome and sequence location of the hypomethylated CpG by CA in promoter regions are compared in Tables 1–5. The details of KEGG pathways that were enriched by hypomethylated promoter regions at 6-h posttreatment are listed in Supplemental Table S3. The results indicate that the global regulatory mechanisms of CA are through demethylation of ^5m^CpG in promoter regions of the key genes involved in calcium channels and calmodulin families.

**Table 1.** Demethylation of ^5m^CpG in promoter regions of calcium signaling pathway

Gene Name	DMR Location in Promoter Region			DMR (Methylation %)			
	Chromosome	Start	End	3 h	6 h	12 h	24 h
*ADRA1A*	chr8	26,722,718	26,723,338	−30.28	−41.25	−30.57	−30.81
*ADRA1B*	chr5	159,343,856	159,343,903	0.00	−33.89	0.00	0.00
*CACNA1D*	chr3	53,529,276	53,529,335	−55.12	−69.06	0.00	0.00
*CALML3*	chr10	5,566,643	5,566,944	−27.06	−47.29	−30.32	−26.91
*CALML4*	chr15	68,498,384	68,498,808	−21.08	−31.79	−21.6	−21.00
*FGF1*	chr5	142,025,495	142,025,537	−54.6	−37.79	−48.58	0.00
*FGF8*	chr10	103,539,541	103,539,685	−19.97	−53.29	−37.87	0.00
*FGFR2*	chr10	123,292,667	123,292,700	−26.28	−47.13	0.00	−24.29
*NTRK3*	chr15	88,749,804	88,749,813	0.00	−49.89	−53.95	−53.82
*PHKG1*	chr7	56,161,011	56,161,115	−15.73	−41.81	−24.54	−33.52
*SPHK2*	chr19	49,127,579	49,128,650	0.00	−60.87	−18.02	−45.29
*VDAC2*	chr10	76,968,663	76,968,696	−27.41	−40.06	−19.71	−26.13

Gene name represent the official gene symbol as recorded in NCBI database. Differentially methylated region (DMR) (methylation %) represents the percentage change in methylation levels induced by cholestenoic acid (CA) treatment at various time points compared with the control group. *P* = 0.0026 at 6 h.

**Table 2.** Demethylation of ^5m^CpG in promoter regions of AMPK signaling pathway

Gene Name	DMR Location in Promoter Region			DMR (Methylation %)			
	Chromosome	Start	End	3 h	6 h	12 h	24 h
*ADRA1A*	chr8	26,722,718	26,723,338	−30.28	−41.25	−30.57	−30.81
*ADRA1A*	chr8	26,722,718	26,723,338	−30.28	−41.25	−30.57	−30.81
*CAMKK2*	chr12	121,713,910	121,713,989	0.00	−37.97	−23.88	0.00
*CPT1A*	chr11	68,610,327	68,610,381	−17.85	−41.69	−16.93	−29.33
*CPT1C*	chr19	50,194,334	50,194,456	−18.80	−34.41	−24.72	−24.09
*CREB3L4*	chr1	153,938,859	153,938,897	0.00	−36.38	0.00	−35.93
*FBP1*	chr9	97,401,415	97,401,438	0.00	−39.18	0.00	−28.62
*LEP*	chr7	127,880,823	127,880,836	0.00	−35.18	−62.7	0.00
*MTOR*	chr1	11,323,660	11,323,854	−20.34	−37.30	−27.13	0.00
*PPARG*	chr3	12,352,022	12,393,324	0.00	−37.32	−27.41	−27.57

Gene name represent the official gene symbol as recorded in NCBI database. Differentially methylated region (DMR) (methylation %) represents the percentage change in methylation levels induced by cholestenoic acid (CA) treatment at various time points compared with the control group. *P* = 0.0008 at 6 h.

**Table 3.** Demethylation of ^5m^CpG in promoter regions of NAFLD signaling pathway

Gene Name	DMR Location in Promoter Region			DMR (Methylation %)			
	Chromosome	Start	End	3 h	6 h	12 h	24 h
*ADRA1A*	chr8	26,722,718	26,723,338	−30.28	−41.25	−30.57	−30.81
*COX7B*	chrX	77,154,454	77,154,461	−30.90	−83.31	0.00	0.00
*COX8A*	chr11	63,740,862	63,741,002	−25.87	−35.30	0.00	−34.15
*LEP*	chr7	127,880,823	127,880,836	0.00	−35.18	−62.70	0.00
*PPARG*	chr3	12,352,022	12,393,324	0.00	−37.32	−27.41	−27.57
*SDHA*	chr5	217,194	217,240	0.00	−37.82	0.00	−23.50
*SDHB*	chr1	17,381,359	17,381,392	0.00	−39.54	−38.35	0.00
*UQCRC1*	chr3	48,647,954	48,648,002	−23.76	−56.12	−23.36	−26.32

Gene name represent the official gene symbol as recorded in NCBI database. Differentially methylated region (DMR) (methylation %) represents the percentage change in methylation levels induced by cholestenoic acid (CA) treatment at various time points compared with the control group. *P* = at 6 h. NAFLD, nonalcoholic fatty liver disease.

**Table 4.** Demethylation of ^5m^CpG in promoter regions of glucagon signaling pathway

Gene Name	DMR Location in Promoter Region			DMR (Methylation %)			
	Chromosome	Start	End	3 h	6 h	12 h	24 h
*CALML3*	Chr10	5,566,643	5,566,944	−27.05	−47.29	−30.32	−26.91
*CALML4*	Chr19	68,498,384	68,498,808	−21.08	−31.79	−21.60	−21.00
*CPT1A*	Chr5	68,610,327	68,610,381	−17.85	−41.69	−16.93	−29.33
*CPT1C*	Chr16	50,194,334	50,194,456	−18.80	−34.41	−24.72	−24.09
*CREB3L4*	Chr5	153,938,859	153,938,897	0.00	−36.38	0.00	−35.93
*FBP1*	Chr7	97,401,415	97,401,438	0.00	−39.18	0.00	−28.62
*LDHA*	Chr21	18,415,186	18,415,286	−29.00	−35.80	−36.43	−32.94
*LDHAL6A*	Chr12	18,475,648	18,476,908	0.00	−38.96	0.00	−22.36
*LDHC*	Chr7	18,433,265	18,433,366	−20.18	−31.45	0.00	−31.77
*PDHA2*	Chr5	96,761,255	96,761,265	0.00	−39.34	−28.93	−38.56
*PDHB*	Chr7	58,420,530	58,420,624	−21.86	−42.31	0.00	−26.74
*PHKG1*	Chr9	56,161,011	56,161,115	−15.73	−41.81	−24.54	−33.52

Gene name represent the official gene symbol as recorded in NCBI database. Differentially methylated region (DMR) (methylation %) represents the percentage change in methylation levels induced by cholestenoic acid (CA) treatment at various time points compared with the control group. *P* = 0.000001 at 6 h.

**Table 5.** Demethylation of ^5m^CpG in promoter regions of chemical carcinogenesis-receptor activation pathway

Gene Name	DMR Location in Promoter Region			DMR (Methylation %)			
	Chromosome	Start	End	3 h	6 h	12 h	24 h
*BCL6*	Chr24	187,455,336	187,455,954	−20.42	−43.30	−33.25	−31.52
*CACNA1D*	Chr7	53,529,276	53,529,335	−55.12	−69.10	0.00	0.00
*CREB3L4*	Chr5	153,938,859	153,938,897	0.00	−36.38	0.00	−35.93
*CYP1A1*	Chr5	75,019,335	75,019,387	−30.07	−55.38	0.00	−27.04
*ESR2*	Chr14	64,751,715	64,751,757	0.00	−33.80	−28.97	−24.69
*FGF8*	Chr10	103,539,541	103,539,685	−19.97	−53.29	−37.87	0.00
*MIR29A*	Chr7	130,562,558	130,562,743	−15.46	−32.98	−22.09	−26.93
*MIRLET7B*	Chr22	46,508,476	46,508,565	0.00	−34.85	0.00	−24.75
*MTOR*	Chr1	11,323,660	11,323,854	−20.34	−37.30	−27.13	0.00

Gene name represent the official gene symbol as recorded in NCBI database. Differentially methylated region (DMR) (methylation %) represents the percentage change in methylation levels induced by cholestenoic acid (CA) treatment at various time points compared with the control group. *P* = 0.0027 at 6 h.

### Effects of CA on the Gene Expression at Transcriptional Level in Human Hepatocytes

**Figure 3.**
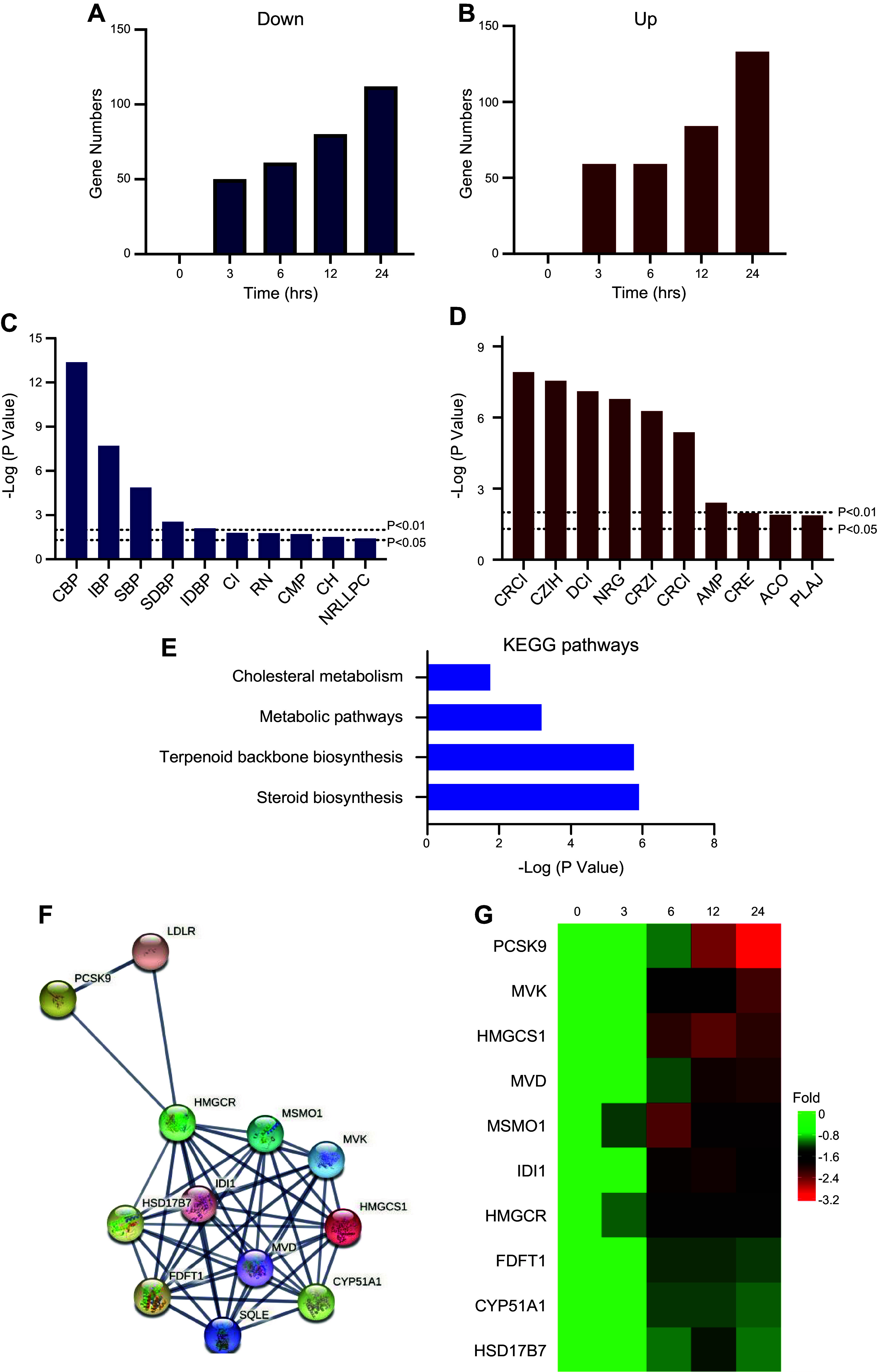
Effect of cholestenoic acid (CA) on transcriptional activities in hepatocytes. HepG-2 cells were cultured in high glucose (HG) medium and treated with 20 µM of CA for 0, 3, 6, 12, and 24 h. *A*: number of downregulated genes regulated by CA. *B*: number of upregulated genes by CA. *C*: top Gene Ontology (GO) terms that are enriched by downregulated genes and treated by 20 µM CA for 6 h. CBP, cholesterol biosynthetic process; IBP, isoprenoid biosynthetic process; SBP, sterol biosynthetic process; SDBP, steroid biosynthetic process; IDBP, isopentenyl diphosphate biosynthetic process, mevalonate pathway; CI, cholesterol import; RN, response to nutrient; CMP, cholesterol metabolic process; CH, cholesterol homeostasis; NRLLPC, negative regulation of low-density lipoprotein particle clearance. *D*: top GO terms that were enriched by upregulated genes following treatment with 20 µM CA for 6 h. CRCI, cellular response to copper ion; CZIH, cellular zinc ion homeostasis; DCI, detoxification of copper ion; NRG, negative regulation of growth; CRZI, cellular response to zinc ion; CRCI, cellular response to cadmium ion; AMP, ATP metabolic process; CRE, cellular response to erythropoietin; ACO, actin cytoskeleton organization; PLAJ, protein localization to adherens junction. *E*: Kyoto Encyclopedia of Genes and Genome (KEGG) pathways enriched by downregulated genes treated by 20 µM CA for 6 h, involved gene numbers were labeled at the end of each bar. *F*: gene-gene network analysis revealed that the downregulated genes are involved in KEGG pathways. *G*: heatmap for the expression levels of downregulated genes that enriched in cholesterol metabolism, metabolic pathways, and steroid biosynthesis pathways. PCSK9, proprotein convertase subtilisin/kexin type 9; MVK, mevalonate kinase; HMGCS1, 3-hydroxy-3-methylglutaryl-CoA synthase 1; MVD, mevalonate diphosphate decarboxylase; MSMO1, methylsterol monooxygenase 1; IDI1, isopentenyl-diphosphate delta isomerase 1; HMGCR: 3-hydroxy-3-methylglutaryl-CoA reductase; FDFT1, farnesyl-diphosphate farnesyltransferase 1; CYP51A1, cytochrome P450 family 51 subfamily A member 1; HSD17B7, hydroxysteroid 17-β dehydrogenase 7.

DNA methylation or demethylation is one of the important mechanisms for regulating gene expression. To examine the effect of CA on whole gene expression in human hepatocytes, mRNA sequencing was used. The results showed that treatment of HepG-2 cells with CA significantly modulated numerous gene clusters. There were 109 different genes (DEGs) regulated by CA (59 were upregulated, 50 were downregulated) at 3-h posttreatment, 120 DEGs (59 were upregulated, 61 were downregulated) at 6 h, 164 DEGs (84 were upregulated, 80 were downregulated) at 12 h, and 245 DEGs (133 were upregulated, 112 were downregulated) at 24 h (Fig. 3, *A* and *B*). The upregulated genes by CA at 6 h are shown in Supplemental Table S4 and those downregulated genes are shown in Supplemental Table S5. To analyze the biological functions of these DEGs, the raw data from 6 h treatment were enriched into the GO and KEGG database. The results showed that 61 downregulated genes were significantly enriched in the lipid biosynthesis process, including cholesterol biosynthetic process (CBP), sterol biosynthetic process (SBP), steroid biosynthetic process (SDBP), cholesterol import (CI) (Fig. 3*C*). While the 59 upregulated genes were enriched into ion process, including cellular response to copper ions (CRCI), cellular zinc ion homeostasis (CZIH), and detoxification of copper ions (DCI) (Fig. 3*D*). The 61 downregulated genes were significantly enriched into four KEGG pathways, namely, steroid biosynthesis, terpenoid backbone biosynthesis, metabolic pathways, and cholesterol metabolism (Fig. 3*E*). The gene networks were constructed by STRING tool (https://string-db.org/) as shown in Fig. 3*F*. The top downregulated genes are list in Fig. 3*G*. The results indicated that CA significantly downregulates key genes involved in steroid biosynthesis pathways in hepatocytes.

### Effects of CA on the Signaling Pathways of Lipid Metabolism

**Figure 4.**
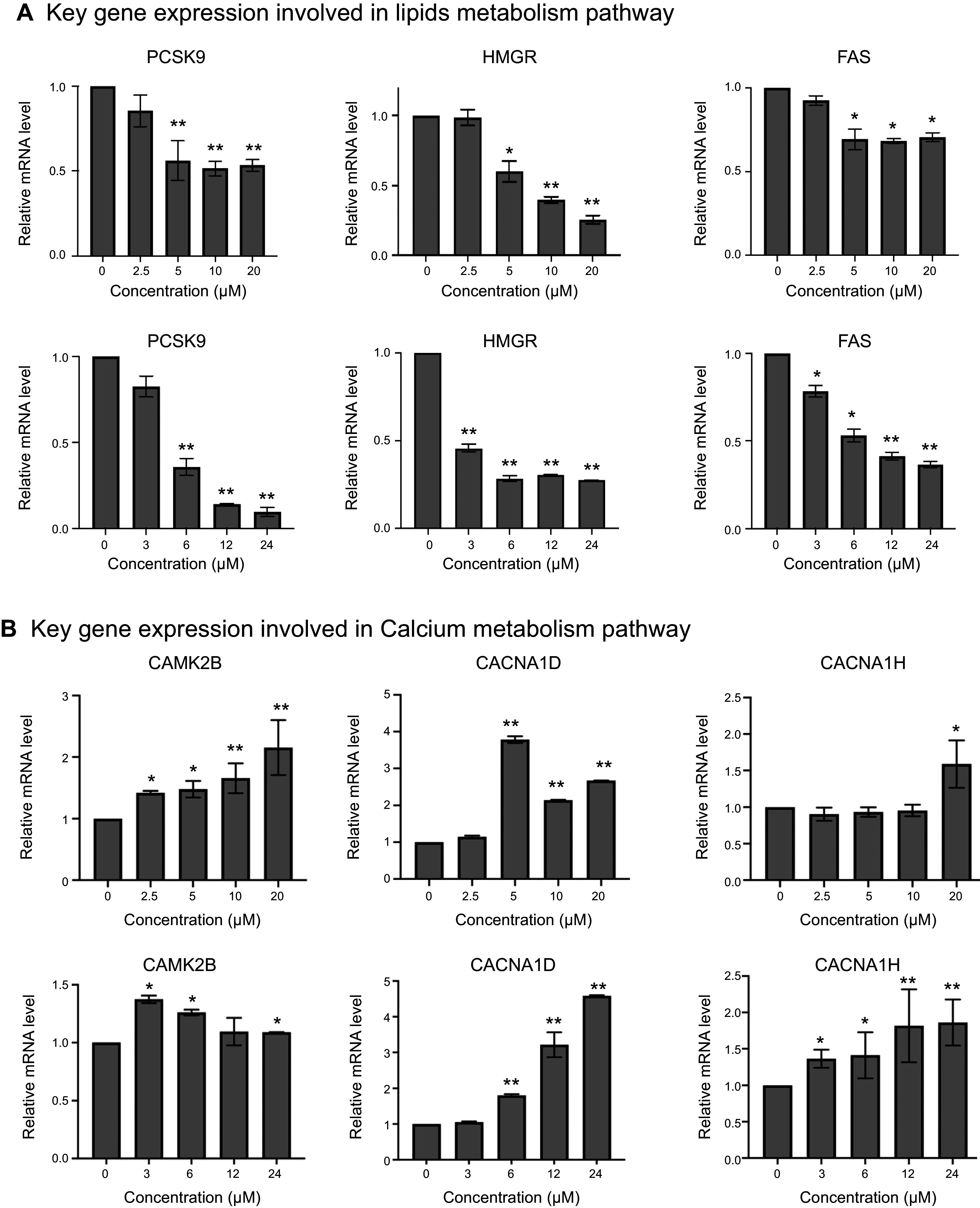
Reverse transcription (RT)-quantitative (q)PCR analysis of gene expression involved in calcium signaling and lipids metabolism pathways. HepG-2 cells were cultured in high glucose (HG) medium for 72 h, followed by treating with 0, 2.5, 5, 10, and 20 µM cholestenoic acid (CA) treatment for 6 h, and 20 µM CA treatment for 0, 3, 6, 12, and 24 h. The expression of key genes involved in calcium signaling and lipids metabolism pathways were measured by RT-qPCR. *A*: dose- and time-dependent expression of key genes involved in lipid metabolism signaling pathway. *B*: dose- and time-dependent expression of key genes involved in calcium signaling pathway. Each bar represents the average data (means ± SD; *n* = 3). *P < 0.05, **P < 0.01.

RT-qPCR was used to confirm the downregulation of selected genes involved in lipid metabolism. HepG-2 cells were treated with 0, 2.5, 5, 10, and 20 µM CA for 6 h, and mRNA levels of key genes involved in lipid metabolism were quantitated by RT-qPCR. The results showed that the levels of mRNA encoding PCSK9, HMGR, and FAS were decreased. The decreases in gene expression were concentration and time dependent as shown in Fig. 4*A*. Previous reports have shown that the calcium signaling pathway regulates lipid and energy metabolism by downregulating gene expression (27, 40, 47, 48). The expression of key genes involved in calcium signaling pathway including calcium voltage-gated channel subunit α1 D (CACNA1D), calcium voltage-gated channel subunit α1 H (CACNA1H) (encoding for calcium voltage-gated channel subunits), and calcium/calmodulin-dependent protein kinase II β (CAMK2B) was quantitated by RT-qPCR. The results showed that all three genes were upregulated in a concentration- and time-dependent manner (Fig. 4*B*). The results indicate that CA may decrease lipid biosynthesis by upregulation of the calcium-AMPK signaling pathway.

### Effects of CA on the Lipid Accumulation in Human Hepatocytes

**Figure 5.**
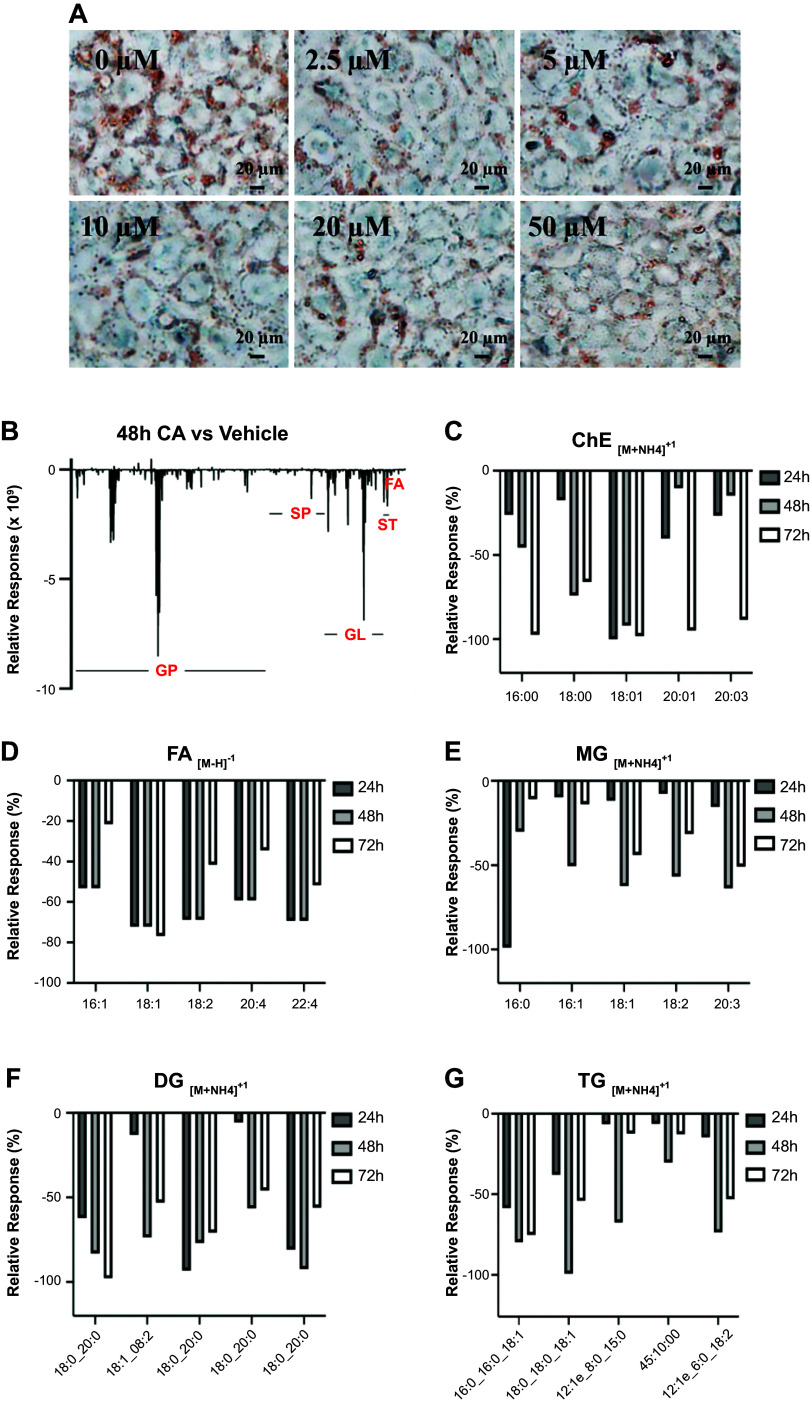
Effect of cholestenoic acid (CA) on lipid accumulation in hepatocytes. *A*: HepG-2 cells were cultured in high glucose (HG) medium for 72 h, followed by treatment of 0, 2.5, 5, 10, and 20 µM CA for another 24 h. The intracellular lipids were stained with 0.3% Oil Red O, *insets* are shown at ×400 magnification of the boxed areas (scale bar, 20 µm). *B*–*G*: lipids levels were measured by untargeted lipidomics assay. HepG-2 cells were cultured in HG medium for 72 h, followed by treatment of 20 µM CA for another 24, 48, and 72 h. Lipids levels were measured by untargeted lipidomics assay. *B*: total lipids relative response of CA vs. vehicle treatment at 48 h. *C*: top decreased ChE (cholesterol ester) lipidlon. *D*: top decreased FA (fatty acid) lipidlon. *E*: top decreased monoglycerides (MG) lipidlon. *F*: top decreased diglycerides (DG) lipidlon. *G*: top decreased triglycerides (TG) lipidlon.

Both WGBS and RNA sequencing results showed that CA may play an important role in lipid metabolism in hepatocytes. To examine the lipid levels in hepatocytes, HepG-2 cells were cultured in HG medium for 72 h, followed by treatment with 20 µM CA for 24, 48, and 72 h. Total lipids were measured by untargeted lipidomics assay. The results showed that CA significantly decreased lipid levels, including glycerophospholipids (GP), sphingolipids (SP), glycerolipids (GL), sterol lipid (ST), and fatty acids (FA). The relative decrease levels at 48 h are shown in Fig. 5*A*; the major decreased lipids were GP. Further analysis for each lipid group showed that the average decreased percentage of cholesterol ester (ChE) at 48 h was 47%, the major lipids decreased lipids are shown in Fig. 5*B*; 65% for FA (Fig. 5*C*), 53% for monoglycerides (MG) (Fig. 5*D*), 76% for diglycerides (DG) (Fig. 5*E*), and 70% for triglycerides (TG) (Fig. 5*F*). The raw data from lipidomic assay from HepG-2 cells treated with CA for 48 h are shown in Supplemental Table S6. The results indicate that CA decreases lipid accumulation via DNA ^5m^CpG demethylation, upregulation of key genes involved in calcium-AMPK signaling pathway, and subsequent downregulation of gene expressions involved in lipid biosynthesis.

### Effects of CA on the Lipid Accumulation in MASLD Mouse Model

**Figure 6.**
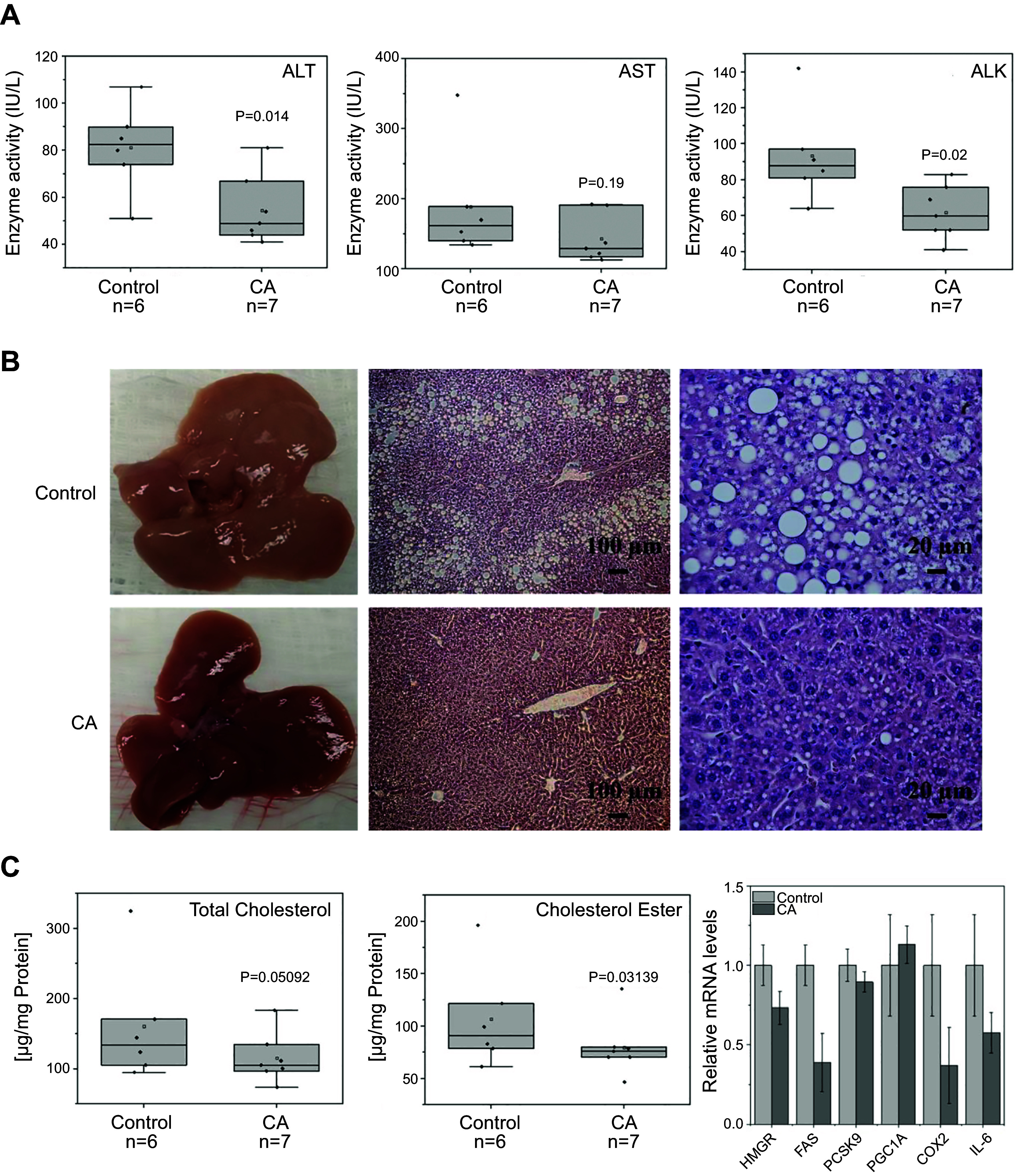
Administration of cholestenoic acid (CA) improves liver function and decreases lipid accumulation in metabolic dysfunction-associated steatotic liver disease (MASLD) mouse model. C57BL/6J female mice were fed a Western diet (TD.88137, Envigo) along with high glucose/fructose water (WDSW) containing 23.1 g/L fructose and 18.9 g/L glucose for 12 wk and followed with 10 mg/kg CA treatment for 3 wk (one dose every other day). *A*: serum was collected and the activities of alanine aminotransferase (ALT), aspartate aminotransferase (AST), and alkaline phosphatase (ALK) were determined by a clinical laboratory. Control represents mice treated with vehicle DMSO injection only. *B*: morphological study of liver tissues treated with vehicle and CA: gross appearance of the livers (*left*); Liver histology at ×100 magnification of the boxed areas (scale bar, 100 µm) (*middle*); at ×400 magnification of the boxed areas (scale bar, 20 µm) (*right*). *C*: effects of CA on the lipid metabolism, the total cholesterol levels (*left*), the cholesterol ester levels (*middle*), and the mRNA levels of key genes involved in lipid metabolism and inflammatory response in nonalcoholic fatty liver disease (NAFLD) mouse model (*right*).

To confirm the role that CA decreases lipid accumulation, MASLD mouse models were used as previously described (49). After 12 wk of WDSW feeding, the mice were treated with vehicle or CA for 2 wk and fasted overnight. Sera were collected for liver function assay and liver tissues were harvested for lipid analysis and gene expression study. As expected, administration of CA alleviates lipogenesis-induced liver injury by significantly decreasing the serum levels of ALT, AST, and ALK levels, 33%, 24%, and 34%, respectively, as shown in Fig. 6*A.* The CA-treated liver tissues became much darker brown than those in the control mice. Examined under microscopy, the CA-treated liver section showed much less lipid droplets than the control section as shown in Fig. 6*B*. Biochemical analysis of lipid compositions showed that CA-treated liver tissues significantly decreased cholesterol and cholesterol ester levels by 25.36% and 28.36%, respectively. RT-PCR analysis showed that mRNA levels involved in lipid biosynthesis were significantly decreased in the CA-treated liver tissues compared with control mice. The results are consistent with those from in vitro hepatocyte models. The study has confirmed the role that CA plays in the maintenance of lipid homeostasis in hepatocytes.

## DISCUSSION

CA, 25HC, and 27HC are synthesized by CYP27A1 in mitochondria (50). The present study reports that CA is a possible unique epigenetic regulator of gene expression. In contrast with 25HC and 27HC, which are potent activators of DNMT1 and silence many genes but do not have any effect on the DNMT3a and DNMT3b enzymatic activities, CA is a potent inhibitor of DNMT3a/b (Fig. 1).

25HC and 27HC are endogenous LXR ligands and play important roles in lipid metabolism, inflammatory responses, and cell survival (51). Recent reports have shown that 25HC and 27HC serve as epigenetic regulators and endogenous activator of DNMT1 (27). High-glucose levels induce lipid accumulation in hepatocytes via generating endogenous 25HC and increasing promotor DNA CpG methylation, subsequently silencing key genes involved in the MAPK-ERK and calcium-AMPK signaling pathways (40). CYP27A catalyzes oxidation of cholesterol in mitochondria and generates 25HC and 27HC. Further oxidation of 27HC by CYP27A produces CA (28). The present study shows that CA appears to have different effects from 25HC and 27HC in regulating DNMT activities: CA upregulates calcium-AMPK signaling pathways and significantly decreases the expression of key genes, including PSCK9, HMGR, ACC-1, and FAS, which are the key elements in cholesterol, fatty acid, and triglyceride biosynthesis, resulting in decreases in lipid accumulation in hepatocytes and improving liver function in high-fat diet-induced MASLD mouse model. The current study has provided evidence that CA may play a preventative role in the development of fatty liver diseases. The regulatory mechanism of CA biosynthesis is unknown. Recent report shows that insulin resistance dysregulates CYP7B1, and substantially increases the CA levels in liver tissue in mouse models of NAFLD. The present results suggest that CYP7B1 may be a key enzyme in regulating CA levels in vivo (52).

CA is a monohydroxy bile acid and has similar chemical properties as sulfated 25HC (25HC3S) and sulfated 27HC (27HC3S) but different chemical structure (31). CA has a carboxy group on the 27-position but no sulfate group as 25HC3S and 27HC3S do. Interestingly, all three molecules inhibit both DNMT3a/b and DNMT1. It is possible that the carboxy group in CA replaces sulfate groups in 25HC3S/27HC3S and the hydroxy group on 3-position in CA replaces the 25- or 27-hydroxy group in 25HC3S/27HC3S. Thus, the unique chemical structure of CA may play a different role from sulfated oxysterols in regulating gene expression.

It is noticed that unlike 25HC3S and 27HC3S, which inhibit DNMT1, CA activates DNMT1 at low concentration and inactivates at high concentration. Furthermore, like 25HC3S and 27HC3S, CA suppresses lipid biosynthesis and decreases lipid accumulation in hepatocytes but does not affect cell proliferation or apoptosis. The current results imply that DNMT1 may be responsible for regulating blocks of genes involved in cell proliferation and cell death, and DNMT3a/b may regulate genes involved in lipid metabolism. Thus, a possible new mechanism, a double preventive mechanism, is implied. When intracellular levels, most likely exogenous cholesterol, are increased, the cholesterol will be delivered into mitochondria, where it is oxygenated to be 27HC and 25HC. 27HC can be further oxygenated to be CA. Interestingly, 27HC and 25HC can be sulfated to be 27HC3S and 25HC3S. All these three molecules, CA, 25HC3S, and 27HC3S are potent inhibitors of DNMTs although CA cannot inhibit DNMT1 at low concentration. Subsequently, they inhibit lipid biosynthesis, suppress inflammatory responses, and antiapoptosis/necrosis, and promote cell survival. These results indicate that hepatocytes have a strong protective mechanism to prevent lipid accumulation and inflammatory responses. Most likely, the generation of CA serves as the first step of preventive function, and sulfation as the second mechanism. However, detailed mechanisms of how DNMTs regulate different gene is currently unknown.

**Figure 7.**
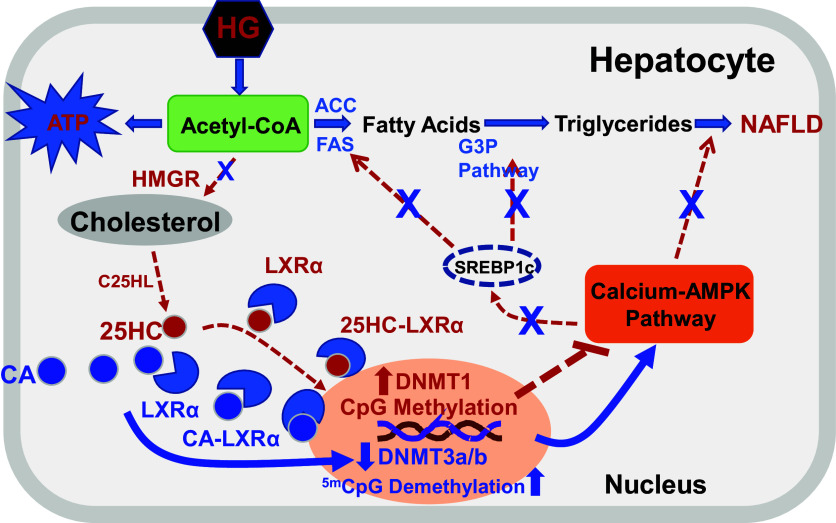
Proposed model of cholestenoic acid (CA) gene regulation in hepatocytes. A high-sugar diet produces excess of acetyl-CoA, which can be used to synthesize cholesterol and long chain fatty acids. Cholesterol is a precursor for the synthesis for 25-hydroxycholesterol (25HC), 27-hydroxycholesterol (27HC), and CA in mitochondria. These oxysterols bind liver X receptors (LXRs) for transport to the nucleus where they regulate genes involved in calcium-AMPK and fatty acid and cholesterol biosynthetic pathways. 25HC, 27HC, and CA may play different roles in the pathophysiology of nonalcoholic fatty liver disease (NAFLD). Dashed red lines represent known pathways, and the blue solid lines represent the proposed pathways regulated by CA.

25HC, 27HC, CA, and other oxysterols have been reported as endogenous LXR ligands (53). Whether these sterol metabolites activate LXRs or LXRs serve as a transporter, delivering their ligands into nuclei, where the ligands regulate epigenomic modification by activating/inactivating epigenetic regulators such as DNMTs, has not been investigated. Recent publications have reported that several cholesterol metabolites including oxysterols, and oxysterol sulfates directly activate or inactivate DNMTs in the nuclei and play opposite role in the gene expression (27). Therefore, it is possible that LXRs may only deliver these molecules into the nuclei, where they regulate gene expression of physiologically linked pathways. The regulation of gene expression by oxysterols through activating/inactivating DNMT enzymatic activity may have an amplifying cascade effect on gene regulation as proposed in Fig. 7.

A recent publication shows that high glucose (HG) levels in culture medium induce lipid accumulation in hepatocytes via epigenetic regulation by 25HC and 27HC (40). The present study shows that addition of CA reverses HG-induced lipid accumulation. Based on these results, we proposed a new regulatory pathway, which may play an important role in the prevention of NAFLD and metabolic syndrome. When cells are incubated in high glucose, sugar consumption will increase intracellular 25HC and 27HC, which in turn will activate DNMT1, resulting in an increase of lipid biosynthesis and lipid accumulation in cells. When intracellular levels increase, CA competes with 25HC and 27HC binding to LXR to enter the nucleus, where CA inhibits DNMT3a/3b and subsequently decreases lipid biosynthesis and lipid accumulation (Fig. 7). The present study suggests that CA can be considered a potential therapeutic target for the treatment of metabolic disorders.

## DATA AVAILABILITY

All data described are contained within the article (Virginia Commonwealth University/McGuire VA Medical Center; Yaping.wang@vcuhealth.org).

## SUPPLEMENTAL DATA

10.6084/m9.figshare.24521338 Supplemental Tables S1–S6: https://doi.org/10.6084/m9.figshare.24521338.

## GRANTS

This study was supported by the United States Department of Veterans Affairs Grant BX003656. S.R. and Virginia Commonwealth University received license-related payments annually from DURECT Corporation.

## DISCLOSURES

S.R. and Virginia Commonwealth University receive license-related payments annually from DURECT Corporation.

## AUTHOR CONTRIBUTIONS

Y.W. and S.R. conceived and designed research; Y.W. performed experiments; Y.W. analyzed data; Y.W. prepared figures; Y.W. and S.R. drafted manuscript; W.M.P., P.B.H., H.-K.M., J.M., M.F., A.J.S., and S.R. edited and revised manuscript; S.R. approved final version of manuscript.
